# Priorities and strategies for improving disabled women’s access to maternity services when they are affected by domestic abuse: a multi-method study using concept maps

**DOI:** 10.1186/s12884-015-0786-7

**Published:** 2015-12-28

**Authors:** Caroline Bradbury-Jones, Jenna P. Breckenridge, John Devaney, Fiona Duncan, Thilo Kroll, Anne Lazenbatt, Julie Taylor

**Affiliations:** School of Nursing, Institute of Clinical Sciences, University of Birmingham, Birmingham, UK; Scottish Improvement Science Collaborating Centre, University of Dundee, Dundee, UK; School of Sociology, Social Policy and Social Work, Queen’s University Belfast, Belfast, UK; Gender Based Violence Nurse Advisor, NHS Fife, Fife, UK; Social Dimensions of Health Institute, University of Dundee, Dundee, UK

**Keywords:** Access, Andersen model, Concept maps, Disability, Domestic abuse, Focus groups, Interpersonal violence, Maternity, Priorities, Strategies

## Abstract

**Background:**

Domestic abuse is a significant public health issue. It occurs more frequently among disabled women than those without a disability and evidence suggests that a great deal of domestic abuse begins or worsens during pregnancy. All women and their infants are entitled to equal access to high quality maternity care. However, research has shown that disabled women who experience domestic abuse face numerous barriers to accessing care. The aim of the study was to identify the priority areas for improving access to maternity services for this group of women; develop strategies for improved access and utilisation; and explore the feasibility of implementing the identified strategies.

**Methods:**

This multi-method study was the third and final part of a larger study conducted in the UK between 2012 and 2014. The study used a modified concept mapping approach and was theoretically underpinned by Andersen’s model of healthcare use. Seven focus group interviews were conducted with a range of maternity care professionals (*n* = 45), incorporating quantitative and qualitative components. Participants ranked perceived barriers to women’s access and utilisation of maternity services in order of priority using a 5-point Likert scale. Quantitative data exploration used descriptive and non-parametric analyses. In the qualitative component of each focus group, participants discussed the barriers and identified potential improvement strategies (and feasibility of implementing these). Qualitative data were analysed inductively using a framework analysis approach.

**Results:**

The three most highly ranked barriers to women’s access and utilisation of maternity services identified in the quantitative component were: 1) staff being unaware and not asking about domestic abuse and disability; 2) the impact of domestic abuse on women; 3) women’s fear of disclosure. The top two priority strategies were: providing information about domestic abuse to all women and promoting non-judgemental staff attitude. These were also considered very feasible. The qualitative analysis identified a range of psychosocial and environmental barriers experienced by this group of women in accessing maternity care. Congruent with the quantitative results, the main themes were lack of awareness and fear of disclosure. Key strategies were identified as demystifying disclosure and creating physical spaces to facilitate disclosure.

**Conclusions:**

The study supports findings of previous research regarding the barriers that women face in accessing and utilising maternity services, particularly regarding the issue of disclosure. But the study provides new evidence on the perceived importance and feasibility of strategies to address such barriers. This is an important step in ensuring practice-based acceptability and ease with which improvement strategies might be implemented in maternity care settings.

## Background

Domestic abuse is the infliction of physical, sexual or mental harm, including coercion or arbitrary deprivation of liberty [1]. A 10-country study on women’s health and domestic abuse reported that between 15 and 71 % of women had experienced physical or sexual violence by their husband or partner [2], and a 28-country European study found that 22 % of all women had experienced physical and/or sexual violence by a partner since the age of 15 [3]. Physical and sexual abuse are associated with emotional harm and abused women are almost twice as likely to experience depression than non-abused women [1]. A growing body of evidence suggests that disabled women are at increased risk of domestic abuse [4–12]. We use the term ‘disabled women’ rather than ‘women with disabilities’, consistent with a social model of disability, which contends that people have impairments but are disabled by social factors [13]. Also, we refer to the United Nations description of ‘disabled people’ as those with “long-term physical, mental, intellectual or sensory impairments which in interaction with various barriers may hinder their full and effective participation in society on an equal basis with others” [14].

Pregnancy is a particularly vulnerable period for domestic abuse among all women, with an estimated 30 % of domestic abuse beginning during the perinatal period [15–18]. Pre-existing abuse may escalate during the twelve months before conception [19] and continue postnatally for up to a year [20, 21]. In contrast, other studies have found that domestic abuse can actually decrease during pregnancy; however it remains a prevalent problem and women with a history of abuse are more likely to see this continue into their pregnancies [22]. Domestic abuse has been linked with adverse foetal outcomes such as premature birth, low birth weight, stillbirth, infection, miscarriage/abortion, placental abruption, and perinatal foetal injury and death [23]. It is associated with significant, adverse maternal health consequences [24–30] and is a principal cause of maternal deaths during childbirth [31]. Paradoxically despite their high risk, many abused women delay accessing maternity healthcare until the third trimester, placing them at greater risk of undetected pregnancy complications and inadequate care [32–34].

Disabled women are vulnerable to pregnancy-related domestic abuse. Sumilo and colleagues [35] found that nearly half of the disabled women giving birth in the UK each year are affected by violence, including experiencing unique forms of abuse related to their disability status. Disabled women have a greater need for services based on the nature and extent of the abuse they experience [12]. But when disability and domestic abuse co-exist, barriers to accessing maternity care are likely to be compounded [5, 9, 10, 12, 36]. Despite this, there has been a lack of attention given to disabled women’s experiences of domestic abuse during pregnancy, and particularly regarding access to maternity services. As a result, the best strategies for achieving universal access to maternity care for this group of women are not fully understood.

### Context of this study

This paper reports on the third stage of a multi-phase study that explored how disabled women’s access to maternity care is affected by domestic abuse. The study took place in the UK during 2012–2014 and comprised three phases: 1) systematic review; 2) individual interviews with women; and 3) focus groups with participants working in maternity care services. Each phase of the study directly informed the next, combining different perspectives in order to achieve a broad understanding of the relationship between disability, domestic abuse, and maternity care access and utilisation. The systematic review [34] highlighted the dearth of literature in this area; only eleven studies were identified as relevant to the topic. The review highlighted the current understanding globally, of the barriers to good maternity care for disabled women experiencing domestic abuse, in particular, mental health problems, poor relationships with health professionals and environmental barriers. Whilst offering partial insight into the problem, the diverse cultural contexts in which studies included in the review took place, and the varied methodological quality of the evidence warranted further in-depth exploration of the topic in order to identify and develop strategies for service improvement.

To better understand the facilitators and barriers to accessing maternity care, phase two involved individual interviews with disabled women affected by domestic abuse who had recent experience of using maternity services. Phase two employed the Critical Incident Technique (CIT) approach [37] asking women to recall and describe specific encounters with health professionals throughout their pregnancy journey; elaborate on the barriers they faced when accessing care; and describe if and how they were able to overcome these barriers. Using a mixture of snowball and convenience sampling we identified five women via organisations such as Women’s Aid to take part in individual interviews in 2013. Collectively, the women reported 45 critical incidents relating to accessing and utilising maternity services. Data analysis was underpinned by Andersen’s model of healthcare use as a theoretical framework (described later) and women’s experiences resonated particularly with the four psychosocial domains affecting access and utilisation of healthcare services: attitudes, knowledge, social norms and perceived control. Women identified positive staff attitude and having control over their own care as the two key factors influencing their decisions about when and how to utilise maternity services. Crucially, the interview findings identified a cyclical process whereby women’s access and utilisation of maternity services is determined by the consequences and outcomes of past healthcare use; if women have negative past experiences they are less likely to use services again in the future.

The third phase of the study reported here, involved asking a range of professionals working in maternity services or supporting women during pregnancy, to consider and respond to the issues women had raised in the phase 2 individual interviews. The aim of this third phase was to explore the extent to which service providers understand the barriers facing women in this situation; identify priority areas for improvement; and develop key operational strategies for facilitating better access to good maternity care for disabled women experiencing domestic abuse.

## Research questions

For disabled women who experience domestic abuse, what are professionals’ perspectives on the:

1. Barriers women face in accessing and utilising maternity services?
2. Priority areas for improving women’s access and utilisation of maternity services?
3. Strategies for improving future access and utilisation?

## Theoretical framework

A clearly articulated theory that is used consistently throughout a qualitative study is deemed to add to its overall quality [38]. As our study was concerned with understanding maternity care access and utilisation, it was underpinned theoretically by reference to the Andersen model of healthcare use [39]. The main premise of the model is that healthcare use is determined by people’s predisposition to use services, their need for healthcare and the enabling and disabling factors that influence their access to care. Andersen [39] has provided a clear distinction between access and realised access to care; where ‘access’ refers simply to the presence of enabling factors and therefore the possibility of getting care, whereas realised access refers to the actual utilisation and receipt of services.

**Fig. 1:**
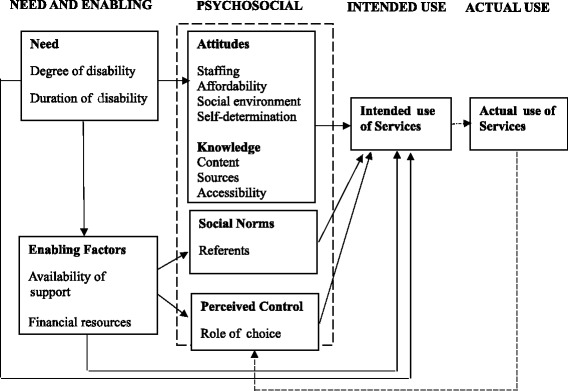
Bradley et al’s [41] revised version of the Andersen Model

Originally developed in the 1960s to explain and predict the factors influencing use of acute services, the Andersen model has been subject to various modifications and revisions since its inception. In fact, a key strength of the Andersen model is that it is flexible to change and adaptation to suit different research topics [40]. Bradley and colleagues [41] criticised the model for ignoring the psychological factors influencing healthcare use and, in collaboration with Andersen, have added an additional four components to the model: attitudes; knowledge; social norms; and perceived control. Anticipating that such factors may be significant for disabled women experiencing domestic abuse, it was this revised version of the Andersen model that we applied to our research (See Fig. 1). As far as we are aware, we are the first to explore its use in the context of maternity care.

## Methods

### Ethics

Ethical approval was granted by the University of Dundee Research Ethics Committee (reference: UREC 12116)]. Participant information sheets were provided to each participant and written consent obtained. Domestic abuse research carries specific ethical challenges around participant safety [42]. So, ample time was offered at the end of each focus group to debrief and address any emotions that had arisen during the discussions. To ensure that the research process was rigorous and ethical, the research team was supported by an advisory panel of researchers, participants and women who had experienced domestic abuse. The advisory panel offered feedback on all stages of the study. Researching a sensitive topic, the research team was also mindful of the need to support one another [43]. Team debriefing, shared reflection and mutual support were essential to ameliorating the potentially distressing effects of collecting and analysing sensitive and upsetting data.

To answer our research questions we used a modified approach to concept mapping. Developed by Kane and Trochim [44], concept mapping is a structured methodology for supporting groups or organisations to co-create their planning and evaluation processes. The key steps in concept mapping methodology include: brainstorming; statement analysis and synthesis; unstructured sorting of statements; multidimensional scaling and cluster analysis; generation of numerous interpretable maps and data displays. Concept mapping is also a general term used to describe the representation of ideas in pictures or maps. In our modified approach we: 1) derived statements from existing literature and women’s interview narratives; 2) asked participants to rate statements; 3) asked participants to carry out an unstructured sorting of statements into groups of similar ideas. Rather than seeing the concept maps as a product, we viewed the concept mapping process as a vehicle for discussion. We were interested in the discussion that participants had whilst sorting the statements, particularly their rationale for valuing certain statements over others. This enabled us to capitalise on the qualitative data as well as the quantitative ranking exercise.

### Recruitment

Participants were recruited via purposeful and simple snowball sampling. An invitation email was sent to GP practices, health visiting and family nurse teams and hospital/community maternity services. Participants were also recruited via the research team’s existing networks, particularly those within midwifery. Having a specialist practitioner from the local health board [FD] on the team facilitated and optimised recruitment.

### Participants

Forty-five participants were recruited to seven focus groups in Scotland during 2014 (See Table 1 for focus group composition). Focus groups took place in both community and hospital settings and the heterogeneity within the sample provided opportunity to compare and contrast the viewpoints of professionals across settings.

**Table 1:** Focus group composition

Focus group	Setting	Participant #
Focus group A	Community	*n* = 12
Focus group B	Community	*n* =3
Focus group C	Community	*n* =11
Focus group D	Hospital	*n* =3
Focus group E	Hospital	*n* =4
Focus group F	Hospital	*n* =9
Focus group G	Hospital	*n* =3
Total		*n* = 45

### Data collection

Data collection within the focus groups was sequential; earlier groups focused primarily on identifying priority barriers to care and latter groups focused on developing improvement strategies. Each of the focus groups began with a short presentation to explain the aims of the study and the findings from the systematic review. Focus groups A-D were presented with a list of barriers to accessing maternity care derived directly from interviews with women in phase 2 of the study. They ranked these individually according to perceived priority on a scale of 1–5 (1 being very high priority and 5 being not a priority) (Table 2). The barriers were also printed on cards to generate discussion within the focus groups. Participants sorted and grouped the priority statements to generate concept maps. During this process their discussions were recorded, generating qualitative data to supplement the quantitative ratings.

**Table 2:** Quantitative rating sheet: barriers and priorities

Barriers to access and utilisation of maternity services	How important is it to address each barrier?				
	1	2	3	4	5
	Very high priority	High priority	Medium priority	Low priority	Not a priority
Fear of disclosure	1	2	3	4	5
Feeling ready to access services	1	2	3	4	5
Misinformation	1	2	3	4	5
Unclear expectations about when and how to access services	1	2	3	4	5
Concealment of abuse from staff	1	2	3	4	5
Being told to ignore what your body is telling you	1	2	3	4	5
Impact of domestic abuse	1	2	3	4	5
Too much information	1	2	3	4	5
Staff not listening to past experience	1	2	3	4	5
Wanting to be seen as ‘normal’	1	2	3	4	5
Unsupportive partner	1	2	3	4	5
Staff being unaware and not asking about abuse and disability	1	2	3	4	5
Staff have all the control	1	2	3	4	5
Baby in control	1	2	3	4	5
Restrictive policies	1	2	3	4	5
Too little information	1	2	3	4	5
Uncomfortable with male staff	1	2	3	4	5
Clarity of communication	1	2	3	4	5
Overwhelmed by too many health professionals	1	2	3	4	5
Judgement from other patients	1	2	3	4	5
Medication overload	1	2	3	4	5
Too much jargon	1	2	3	4	5
Fear of judgement from staff	1	2	3	4	5
Societal stigma	1	2	3	4	5
Hyper alertness in verbal and non-verbal interactions	1	2	3	4	5

In groups D-G, participants were presented with the priority barriers derived by the earlier focus groups and were asked to identify strategies to address these areas. (There was overlap in group D, where participants completed both rating exercises). Participants in focus groups D-G were presented with a list of strategies identified by women participants in phase 2 of the study (using the approach described earlier). They rated the strategies in order of importance and ranked the feasibility of each using a 5-point Likert scale (Table 3). Strategies were also printed on cards to facilitate group discussion. This served to consolidate the existing list of strategies, identify operational processes, and generate new strategies.

**Table 3:** Quantitative rating sheet: strategies and feasibility

Strategies for enabling better access to maternity care	How would you prioritise these strategies? How feasible would it be to put this in place?									
	1	2	3	4	5	1	2	3	4	5
	Very high	High	Medium	Low	Not a priority	Very feasible	Feasible	Some challenges	Very challenging	Impossible
A ‘preferred contacts’ screening list	1	2	3	4	5	1	2	3	4	5
Staff who understand complex needs	1	2	3	4	5	1	2	3	4	5
Access to specialist disability support	1	2	3	4	5	1	2	3	4	5
More frequent appointments	1	2	3	4	5	1	2	3	4	5
Send out appointment reminders	1	2	3	4	5	1	2	3	4	5
Non-judgemental staff attitude	1	2	3	4	5	1	2	3	4	5
Staff listen to women’s past experiences	1	2	3	4	5	1	2	3	4	5
Staff being supportive and available	1	2	3	4	5	1	2	3	4	5
Seeing services closer to home	1	2	3	4	5	1	2	3	4	5
Women coming prepared for appointments	1	2	3	4	5	1	2	3	4	5
Family support	1	2	3	4	5	1	2	3	4	5
‘Having a laugh’ with staff	1	2	3	4	5	1	2	3	4	5
Trusting relationship with practitioner	1	2	3	4	5	1	2	3	4	5
Negotiate care decisions with women	1	2	3	4	5	1	2	3	4	5
Access to specialist domestic abuse support	1	2	3	4	5	1	2	3	4	5
Childcare arrangements to let women attend	1	2	3	4	5	1	2	3	4	5
Provide information about domestic abuse to all women	1	2	3	4	5	1	2	3	4	5
Accessing a formal/informal support group	1	2	3	4	5	1	2	3	4	5
Finding ways to see women without their partner	1	2	3	4	5	1	2	3	4	5
Signposting and referral to other sources of help	1	2	3	4	5	1	2	3	4	5
Demystify the role of social services	1	2	3	4	5	1	2	3	4	5
Staff training in mental health issues	1	2	3	4	5	1	2	3	4	5
Clear and appropriate information giving	1	2	3	4	5	1	2	3	4	5

### Data analysis

Quantitative data exploration used descriptive and non-parametric analyses for ratings of barriers and feasibility of strategies. Qualitative data were analysed inductively using Ritchie and Spencer’s [45] approach to framework analysis; involving sifting, charting and sorting the data into key themes. Two researchers (CB-J & JPB) undertook the analysis independently and then met to discuss and agree the emerging themes. These were then mapped to Andersen’s theoretical framework, applying a principle of the ‘closest fit’. For example, themes relating to strategies for improvement were classified as ‘enabling factors’ and because the framework does not refer to ‘barriers’ *per se*, we mapped our identified barriers to the ‘needs’ dimension of the model (Fig. 1).

## Results

### Quantitative results

In total, 45 professionals were included in seven focus groups. Twenty-nine participants ranked and discussed the barriers. Eighteen participants took part in the discussion of strategies. Two participants completed both exercises. Table 4 gives a breakdown of participant characteristics.

**Table 4:** Participant characteristics

Participants discussing barriers (*n* = 29)		Participants discussing strategies (*n* = 18)	
Profession	n (%)	Profession	n (%)
Criminal Justice Assistant	1 (3.4)	Midwife	15 (83.3)
Health Visitor	9 (31.0)	Nursing Assistant	1 (5.6)
Medical Student	2 (6.9)	Student Midwife	1 (5.6)
Midwife	10 (34.5)	Maternity Care Assistant	1 (5.6)
Nurse	4 (13.8)		
Nursery Nurse	1 (3.4)		
Psychologist	1 (3.4)		
Social Worker	1 (3.4)		
Setting		Setting	
Community	19 (65.5)	Community	7 (61.1)
Criminal Justice	2 (6.9)	Hospital	11 (38.9)
Hospital	8 (27.6)		
Years of experience working in maternity services		Years of experience working in maternity services	
0–10 years	8 (27.6)	0–10 years	5 (27.8)
11–20 years	3 (10.3)	11–20 years	3 (16.7)
21–30 years	13 (44.8)	21–30 years	7 (38.9)
31 and more years	5 (17.2)	31 and more years	3 (16.7)

The perceived barriers to women’s access and utilisation of maternity services and their ranking in terms of priority are shown in Table 5. Tables 6 and 7 show the list of strategies to address these barriers, ranked in order of importance and feasibility. In all tables, ranking was established using mean scores and standard deviation. Some items have shared ranks, with standard deviation being used to differentiate between shared means where possible. The highest ranked barriers were: staff being unaware and not asking about domestic abuse and disability (M 1.34 SD 0.553); impact of domestic abuse on women (M 1.36 SD 0.559); and women’s fear of disclosure (M 1.41 SD 0.780). There were no significant differences in relation to barrier ratings based on the number of years worked in maternity services. However, there were differences in the ratings of barriers between those who had worked with this population of women (disabled women affected by domestic abuse) before and those who had not. They differed on the following items ‘Fear of Disclosure’ (Fisher Exact Test 7.103, Exact Significance (2-sided) *p* = 0.025); ‘Staff not listening to past experiences’ (Fisher Exact Test 9.977, Exact Significance (2-sided) *p* = 0.004); ‘Judgement from other patients’ (Fisher Exact Test 7.076, Exact Significance (2-sided) *p* = 0.048), and ‘Societal Stigma’ (Fisher Exact Test 6.132, Exact Significance (2-sided) *p* = 0.035). The top two priority strategies were identified as: 1) providing information about domestic abuse to all women (M 1.22 SD 0.428); 2) promoting non-judgmental staff attitude (M 1.22 SD 0.488). These were also considered to be amongst the most feasible strategies to implement in practice: providing information about domestic abuse to all women (M 1.44 SD 0.784); promoting non-judgmental staff attitude (M 1.61 SD 0.788).

**Table 5:** Perceived Priority of Barriers (*n* = 29)

Rank	Type	Mean	SD
1	Staff being unaware and not asking about domestic abuse and disability	1.34	.553
2	Impact of domestic abuse on women	1.36	.559
3	Women’s fear of disclosure	1.41	.780
4	Concealment of domestic abuse from staff	1.43	.690
5	Fear of judgment from staff	1.52	.574
6^a^	Clarity of communication	1.52	.688
6^a^	Staff not listening to past experiences	1.52	.688
7	Unclear expectations about when and how to access services	1.55	.506
8	Societal stigma	1.66	.614
9	Unsupportive partner	1.66	.769
10	Being told to ignore what your body is telling you	1.69	.604
11	Hyperalertness in verbal and non-verbal interactions	1.69	.660
12	Wanting to be seen as normal	1.72	.649
13	Overwhelmed by too many health professionals	1.76	.689
14	Feeling ready to access services	1.76	.786
15	Misinformation	1.79	.620
16	Uncomfortable with male staff	1.86	.833
17	Staff have all the control	1.89	.916
18	Too little information	1.96	.706
19	Too much information	1.97	.731
20	Judgment from other parents	2.00	.816
21	Too much jargon	2.07	.799
22	Medication overload	2.07	.884
23	Restrictive policies	2.14	.756
24	Baby in control	2.32	1.02

^a^Shared rank

**Table 6:** Importance of strategies (*n* = 18)

Rank	Type of strategy	Mean	SD
1^a^	Provide information about domestic abuse to everyone	1.22	.428
1^a^	Non-judgmental staff	1.22	.488
2	Access to specialist domestic abuse support	1.24	.437
3 +	Staff being supportive and available	1.28	.461
3 +	Clear and appropriate information given	1.28	.461
3 +	Finding ways to see women without their partner	1.28	.461
3 +	Signposting and referral to other sources of help	1.28	.461
4	Trusting relationship with practitioner	1.28	.575
5	Staff listen to women’s past experiences	1.44	.511
6	Asking about abuse and knowing what happens next	1.50	.618
7	A preferred contacts screening list	1.61	.502
8	Access to specialist disability support	1.65	.786
9	Demystifying the role of social services	1.72	.575
10	Staff who understand complex needs	1.78	.808
11	Staff trained in mental health issues	1.83	.786
12	Accessing a formal/informal support group	1.94	.680
13	Family support	2.00	.686
14	Women coming prepared for appointments	2.22	.878
15	Child care arrangements to let women attend	2.29	.849
16	More frequent appointments	2.29	.920
17	Send out appointment reminders	2.53	.943
18	Having a laugh with staff	3.00	1.138

^a^ + shared ranks

**Table 7:** Feasibility of strategies (*n* = 18)

Rank	Type of strategy	Mean	SD
1	Provide information about domestic abuse to everyone	1.44	.784
2	Staff listen to women’s past experiences	1.50	.707
3	Non-judgmental staff	1.61	.778
4	A preferred contacts screening list	1.67	.840
5	Clear and appropriate information given	1.72	.669
6	Signposting and referral to other sources of help	1.83	.618
7	Staff being supportive and available	1.88	.697
8	Negotiate care decisions with women	1.88	.781
9	Asking about abuse and knowing what happens next	1.89	.832
10	Having a laugh with staff	2.00	.555
11	Trusting relationship with practitioner	2.00	.707
12	Finding ways to seeing women without their partner	2.06	.802
13	Staff who understand complex needs	2.17	.786
14	Send out appointment reminders	2.17	.985
15	Access to specialist disability support	2.18	.883
16	More frequent appointments	2.28	.895
17	Women coming prepared for appointments	2.31	.873
18	Access to specialist domestic abuse support	2.39	.979
19	Demystifying the role of social services	2.50	.985
20	Accessing a formal/informal support group	2.63	.957
21	Staff training in mental health issues	2.67	.840
22	Seeing services closer to home	2.71	.588
23	Family support	2.76	.831
24	Child care arrangements to let women attend	3.28	.958

## Qualitative findings

Qualitative data were generated in tandem with the quantitative ranking so while analysed inductively, it is unsurprising that qualitative findings map closely to the quantitative results. The dominant themes from the qualitative data relate to issues of awareness and disclosure.

### Understandings and awareness of disability and domestic abuse

Participants in the study reported that they sometimes lack awareness of how to deal with the combined issues of disability and domestic abuse among women in their care. For example, they reflected that women may not perceive themselves as disabled and may therefore withhold particular information about their impairment or health condition. This could also be impairment specific, with participants finding mental health much more ambiguous and difficult to broach than physical or sensory impairments:

“Asking about mental health generally is really hard. If you are doing an antenatal assessment and you are asking “have you had any mental health problems?” it’s quite difficult. A lot of the girls that I see, if you were talking about ‘mental health issues’ they would say no, because they wouldn’t really understand what that is. Whereas if you said, “have you ever had depression?” or “have you ever had panic attacks?” they might say yes. And a lot of people don’t see depression as a mental health issue or disability as such.” (B)

Some midwives felt that they lacked understanding of mental health issues:

“In my experience, I know I have found it difficult… a lot of the midwives now, including myself, have been direct entry [into midwifery] so we don’t have any experience in general or mental health nursing.” (E)

Our analysis also hints at a lack of understanding and prevailing stereotypes about learning disability. It also shows the complexity of providing maternity care for women where disability and domestic abuse co-exist.

“People who are very intelligent can be bad parents and maybe we should be supporting people with learning disabilities to be parents because, you know, they are very warm and loving people who will support a child… I’ve got a woman coming in with learning disabilities, she is with a partner we have known to be violent to somebody else… it’s so complex.” (F)

In relation to domestic abuse awareness, participants stated that training was helpful but much of their knowledge had been generated through experience:

“Knowing to pick up an atmosphere, pick up on non-verbal cues etcetera that only comes with experience. You might miss things in one situation but pick up on them in another. You wouldn’t get that in a week’s training.” (C)

Despite their training and experience, however, the language that some health professionals used still belied some persistent stereotypical views about abused women:

“They’ve kept themselves in this situation, they’ve not walked away from the situation and so immediately her baby is at risk and we are going to have to deal with it… The situation never goes away until they move onto somebody else and get into the same situation again, and again, because they just pick the same partners all the time.” (F)

“Some people might like their man being completely in charge of everything, maybe a more traditional wife, you know.” (G)

Participants reflected that their ability to identify and respond to domestic abuse is also impacted upon by women’s own awareness of their situation. Women may not always recognise their experiences as abusive, particularly if the abuse does not involve physical violence. They felt that this was compounded by a general lack of understanding about domestic abuse within society and that not all people would relate to the term or understand it in the same way:

“She might think, “it’s not domestic abuse, he doesn’t hit me”.” (C)

“When a person experiences emotional or psychological abuse, they might not recognise their experiences as domestic abuse.” (A)

Participants reported that the nature of a woman’s impairments could have an impact on her insight into the abusive situation. The following quote captures this issue:

“if a woman is disabled, if she has like a learning disability or mental illness or something like that, it’s a lot more difficult for them. Like they have difficulty articulating themselves or they don’t understand cognitively how that’s [domestic abuse] impacting on them. I’ve worked with a lady with learning disability – she couldn’t understand it was domestic violence, because of her learning difficulties. She just accepted that it was the way it was, to live with someone who is controlling them… how people interact with you in that sort of punitive, chastising way that she thinks is normal.” (C)

### Fear of disclosure among women and professionals

Participants characterised women by fear: fear of their partner, fear of the consequences of disclosing and fear of the unknown, that is, what happens to them, to their children, and their partner if services ‘find out’. There was consensus amongst all focus groups that women’s fear of disclosure was the most significant barrier to optimum maternity care. This is reflected in the quantitative data, where fear of disclosure is one of the top rated barriers. Participants identified that fear of disclosing may account for women in their caseloads who are:

“Very rarely seen, trying to mask what their lives are like. How do we reach those women who know they are pregnant but have not accessed services yet?” (C)

They explained:

“As soon as women seek help, they are afraid that social services will get involved and they will be found incapable of taking care of themselves or their baby. Domestic abuse is a trigger for child protection so you can understand why people might not divulge.” (D)

“Fear of social services is huge. They tell you about domestic abuse and it’s a child protection issue, its unborn baby protocol, its social services and they think ‘God, they’re going to take my baby away.”

“Yeah, and it snowballs and then they are unlikely to tell anyone again.” (C)

Participants perceived that non-disclosure is a considerable barrier to good maternity care. If women do not disclose their situation fully (either their disability, domestic abuse or both), participants believed that they cannot tailor optimal care to suit women’s needs effectively:

“If we know about the problem, and the woman is willing to share with us, there is a whole raft of extra support that can be put in place to make sure that her care is tailored specially for her.” (F)

The priority for participants was thus to “make disclosure easier” for disabled women experiencing domestic abuse by removing psychosocial and environmental barriers.

Paradoxically, professionals are also afraid of disclosure. Participants in this study highlighted that it can be difficult to find the right words to ask about domestic abuse and disability. In particular, they are wary of offending women and their partners. This fear often centred round challenging women’s “normality”:

“How do you say what they have as ‘normal’ is actually not normal. Who are we to judge what’s normal and what’s not. It’s normal to them. It’s wrong to us but that’s the way it is to them. It’s very difficult to turn round to women and say actually that’s not normal.” (C)

Although participants found asking questions about abuse difficult, they highlighted a far greater difficulty with responding to a positive disclosure. They are not always sure about the correct processes following disclosure and how to react to an affirmative answer:

“I don’t know how you instil confidence in asking about it. Maybe it’s experience; it’s your gut instinct. But if you ask that question, you are going to have to deal with it and it’s difficult for people.” (A)

Anxiety about how to respond to disclosure was further heightened for participants who had never experienced a woman disclosing abuse and saying ‘yes’:

“You see I’ve never come across anybody actually saying there is an issue. And I try to say in a serious way that we can help you, or we are there if you need us in the future…I don’t have a fear, but I hope that if I’m in that position that I’ll make them feel comfortable so in time they may indicate that they need help. But I don’t know. I don’t always know the right thing to actually say.” (G)

### Environmental barriers to disclosure

Participants identified barriers within the physical environment that prevented women from accessing maternity services. Disabled women are often labelled ‘high risk’ at the start of their pregnancies, meaning that they tend to see more healthcare professionals than non-disabled women. This is in addition to the health and social care professionals that are likely already involved in their lives. While this can sometimes be essential and appropriate, travel to multiple appointments can be difficult for women with a physical impairment or health condition. For disabled women who also experience domestic abuse, there are compounding difficulties:

“Most women go straight to the midwife now when they know they are pregnant but if you have a disability or a long term health condition straight away you become a consultant’s…This mother I’ve got has quite a long medical history and is quite debilitated with it. Add that to being pregnant, so that’s upped the ante with her visits to the physician, 25 miles away. And it’s not joined up care. It’s not seeing the physician and the obstetrician [on the same day], it’s seeing one this week and one the next. That’s difficult, let alone having a partner who wants to know where you are, why you are away and he’s got the money, the finances that take you there. Or he’s saying, “I’m not looking after the children” and she’s thinking what will happen if I’m not there.” (B)

Participants also identified the impact of the physical environment on disclosure. There was consensus across all seven focus groups that difficulty seeing women alone, without the presence of her partner or family member, was a significant barrier to disclosure:

“Trying to get to see women on their own whether they have a disability on top of the domestic violence or not. Just getting to see women on their own is the biggest barrier.” (F)

“It can be difficult to explore issues fully when the partner is there. Some partners are clearly controlling, you see it all the time, the person you are doing the assessment for isn’t getting a chance to speak.” (A)

“Whenever you went to the house, he would never leave the room. Never. Very rarely was she alone.” (B)

Depending on the physical location of the appointment, participants found it easier or more difficult to negotiate seeing women alone. This is demonstrated in the following exchange between participants working in the labour ward, the community and the antenatal clinics:

“We’ve become better at that [seeing women alone] because we’ll take them off to do their height and weight…“Exactly, but you’ve got maybe more opportunity to do that [in the hospital] than we’ve got in community. We’ve got nowhere else to go with her” “Although, for us in the antenatal clinic, all the equipment is in room so we can’t do that either.” (C)

In participants’ experience, asking partners to leave is difficult in all situations. They feel awkward and lack confidence in asking to see disabled women on their own and have particular difficulty in distinguishing between a caring partner and an abusive one:

“I’m thinking of one particular woman. She’s had about six or seven pregnancies and he is in charge of everything. He comes to the desk, he tells us her name… although we’ve never had any proof that there is domestic abuse going on. But that lady is somebody who is disabled so you know, maybe he’s caring but maybe there is [abuse]. We’ve always suspected there is something there because of his super-controlling attitude.” (G)

Speaking to women alone is even more difficult if they have literacy difficulties or communication impairment. Often in these cases, participants used family members or partners to support communication, however, this is also problematic:

“I’m thinking about the huge number of women we’ve got with dyslexia. They don’t need diagrams – they need the things read to them. That’s what I would normally do with them. Or I usually say, is there someone who you usually read things with?” – “It might be her partner” – “oh, I’d better stop doing that then. I’ll read them that leaflet.” (E)

Participants frequently talked about using written communication when it was not possible to speak to women alone, for example handing women a leaflet about domestic abuse or passing a written note to ask secretly, “are you safe for me to leave you alone today?” These strategies for overcoming the difficulty of seeing women alone, while useful for many women, were only suitable for women who could read, understand and respond in this format. Two focus groups identified that they did have alternative communication aids, for example easy read and pictorial information sheets, however they stated that these had been forgotten about and were rarely used.

Overall, participants in the study discussed a range of issues that influence access to optimum maternity care for disabled women who experience domestic abuse. However they were also able to identify a range of strategies that might address these that hinge principally around demystifying disclosure and creating physical spaces to facilitate disclosure.

### Demystifying disclosure

Recognising women’s fear of disclosure as a significant barrier to optimal care, participants questioned how they could make the process “more transparent and less scary” (C). There was agreement that women need greater clarity about what happens following disclosure:

“Instead of just asking the question about domestic abuse, you might also want to be talking in terms of confidentiality etcetera, and be clearer about what we do with their information. We don’t go into that as fully as we should. We’d have to give more of a preamble before you launch into the question about domestic abuse. The questions are all there but I don’t say to everybody that if they tell me something, it has to go somewhere.” (C)

Participants all agreed that giving women more information about the disclosure process before asking the question gave them opportunity to weigh up the pros and cons before deciding to disclose. Participants felt this might afford women a greater sense of control and hence encourage more women to disclose. Conversely, participants also warned of the potential dangers of giving too much information which could potentially have the opposite effect:

“You would need to get the balance right, so not to overwhelm them.” (D)

The only way to facilitate informed disclosure is to support staff in making informed enquiries. Participants reported that they need to understand the disclosure process and the potential consequences arising from disclosure. This extends beyond simply having the confidence to ask, but knowing how to respond to a positive disclosure; knowing the next steps and having a clear pathway to follow. There are however some potential pitfalls in this approach:

“The disclosure process isn’t universal. You can’t give them [women] any definites [promises] – what if they disclose something you are not prepared for? If women are overloaded with all the potential options for what might happen, they might be scared away from services and choose not to disclose.” (E)

### Creating physical spaces to facilitate disclosure

Three focus groups gave an example of current practice for facilitating disclosure through environmental cueing. All women are required to give a urine sample, providing a small window of opportunity when women will be alone without their partner. Participants described a system in which:

“There are red sticky dot labels and an information sheet on the back of the [toilet] door which says “if you are afraid of your partner, experiencing domestic abuse etcetera and want to speak to a midwife on their own please put a red sticker on your urine container”.” (F)

Participants also suggested that having posters about domestic abuse in the hospital or clinic environment could convey the message to women that “we talk about it, we know about it, we understand it” (A). Again, however, these strategies rely on written information that may not be suitable for women with visual or communication impairments.

To overcome difficulties travelling to different appointments (e.g. psychologist) and other agencies (e.g. Women’s Aid) participants thought it would be ideal to have a ‘one stop shop’ where all services were in the same place. They felt that this would be more convenient for women and reduce the number of missed appointments.

“It would be great just to say, “there is somebody there for you just now – just go down the corridor”. It might encourage women to come forward and divulge information if they knew there was somewhere they could go right there and then.” (F)

Another suggestion was to hold a Women’s Aid drop in within the antenatal clinic. This would provide better support for women and participants also felt that it would create a safe ‘cover’ for their domestic abuse support:

“They could tell their partners they were just going to the hospital, rather than having to explain to their partner they are travelling to the Women’s Aid.” (F)

Participants identified that women experiencing domestic abuse tend to make more unscheduled, emergency appointments than women who do not experience domestic abuse. They identified that, rather than simply recording this in the notes as is current practice, there should be a system put in place for monitoring the number of emergency appointments and responding to this:

“They are back and forth quite a lot with trivial things. They come in for triage then get sent away again. It’s difficult to say for sure, but it’s a feeling. You do see some girls and this is their only pregnancy, and their notes are really quite thick because they have come through triage so many times.”“It’s not the first time you’ve seen in the notes the doctor will write ‘30 admissions’ or ‘10 admissions’ and I don’t think anyone ever really follows it up.” (D)

Numerous unscheduled appointments provide an opportunity to identify and follow up on potential issues, specifically domestic abuse but also including issues such as mental health problems or previous pregnancy loss. Participants identified this as one strategy for facilitating early identification of additional needs to ensure that maternity care can then be individually tailored. However, there may still be barriers to disclosure if women are unwilling to disclose or if practitioners cannot see women alone. Participants perceived that consistent contact with the same health professional could facilitate familiarity and trust, therefore increasing the likelihood of disclosure. Although it was noted that this was not always possible, particularly in busy clinics, participants identified the importance of maximising consistency.

Overall, our findings highlight the importance of facilitating optimal opportunities and a safe environment for women to disclose. Demystifying the disclosure process, providing the right space and developing a non-judgemental, trusting relationship are essential strategies in ensuring that women receive optimal care. Our findings demonstrate, however, that there is still a lack of understanding about how to make disclosure easier for disabled women and the intersection between disability and domestic abuse remains poorly understood.

## Discussion

Disclosure is a well-recognised concern for both disabled people [46] and those who experience domestic abuse [47–49]. This was a clear finding in our study, with professionals identifying that women’s fear of disclosure is a significant barrier to optimum maternity care. Participants in all focus groups talked about the misconceptions surrounding the impact of media portrayal of social workers as functioning solely to remove children into care. They talked of a pervasive fear among women: fear of their partner, fear of the consequences of disclosing and fear of the unknown. This aligns with previous research [50–53] and could account not only for lack of disclosure but also avoidance of services altogether. The findings of our study show how the experience of domestic abuse can in itself constitute a considerable barrier to accessing services. In order for women to disclose, they need to have an understanding and awareness of their situation. Similarly, previous studies have shown how the psychological impact of abuse is such that it skews women’s perceptions of their situation, where they fail to recognise the abusive nature of their experiences [53]. Whilst fear of disclosure could apply to all women, in the previous phase of this study [54] we identified that disabled women experiencing domestic abuse face the fear of double disclosure; they worry not only about the stigma surrounding domestic abuse but also about how health professionals’ misconceptions about disability may affect their care.

Evidence suggests that disabled women are subjected to prejudiced beliefs about their ability to be ‘good mothers’, especially within the medical world where they are classified as ‘high risk’ [55]. Although more recent evidence suggests that disabled women’s experiences of maternity care might be improving, women with mental health issues, learning disabilities and multiple disabilities still report particularly poor access to maternity care [56]. Participants also reported that the presence of abusive partners had an impact on their ability to provide optimal care, such as their partners being present at appointments, making it difficult to see women alone. This issue was particularly significant for community participants, where it was especially challenging to ask partners to leave the room when visiting women in their own homes. Moreover, participants felt awkward and less confident asking to see disabled women on their own and they had a particular difficulty in distinguishing between a caring partner, and an abusive one. Indeed, this has been identified as a significant barrier to disclosure for disabled women, who worry that they will not be believed because their abusive partner is also their main carer [12].

A scoping study of domestic violence interventions in primary and maternity care settings in seven European countries [57] identified that health professionals use a variety of approaches to identify domestic abuse. However, our focus group participants still expressed anxieties about the disclosure process. In particular, they were afraid of offending women and their partners. This is again reflected in other literature, which shows that lack of confidence among staff in relation to abuse is underpinned primarily by fear of offending women or knowing how to respond appropriately post-disclosure [53, 58]. In previous studies, we have referred to this as ‘opening a can of worms’ [53, 59]. From the current study it is clear that staff need to understand the disclosure process and the potential consequences arising from disclosure. This extends beyond simply having the confidence to ask, but knowing how to respond to a positive disclosure; knowing the next steps and having a clear pathway to follow. Participants identified a need for greater clarification of this process both for themselves and for women in their care.

When individuals are fully informed and know what to expect from their healthcare, they are more likely to use services [60]. Informed decision making is fundamental to effective birth planning and the empowerment of women in childbirth [61]. National Institute for Health and Care Excellence (NICE) guidelines [62] offer best practice advice on the care of women in labour and the principles of good care can be extrapolated to other maternity contexts. The guidelines highlight the importance of accessible information that takes into account any additional needs of women such as physical or cognitive disabilities. This has direct relevance to the findings of our study. Effective interdisciplinary teamwork and integration across maternity care settings is also important [63]. Again, in the context of the study, initiatives such as the one-stop shop might be useful, but they require communication and co-operation across services.

Focus group participants highlighted the importance of engaging with women in a non-judgmental way that takes account of the emotional consequences of domestic abuse. Non-judgmental attitude is important in facilitating the disclosure process [53]. The findings of this study support those of earlier research, where health professionals – including midwives – have been found to hold fixed, stereotypical views of domestic abuse, including the view that many women are complicit in their own abuse [59]. A comprehensive, global analysis of the contribution that midwifery can make to the quality of care of women and infants highlighted the importance of strengthening women’s capabilities in the context of respectful relationships [63].

Personal views about health services and care providers have direct impact on intended use of services [41]. Anticipation of poor relationships with health professionals has been found to be a critical barrier to accessing care [64, 65]. It is therefore essential that every participant-service user interaction is positive and non-judgmental, taking all available opportunities to change women’s negative perceptions of maternity care which are often a barrier to utilising services [34]. In midwifery, the development of woman-centred care over the past few decades has sought to empower women and strengthen the quality of their care by involving women as active partners in decision making and respecting women’s preferences [62, 66, 67]. The only way to ensure that care remains specific to the individual woman is to encompass the foundations of woman-centred care [61]: working with women as partners; respecting their expertise; and making decisions based upon individuals rather than stereotypes. All these things situate women in a context of control rather than disempowerment. They resonate with the findings of this study and are important considerations in ensuring optimum maternity care for disabled women who experience domestic abuse.

### Study strengths and limitations

While every effort was made to ensure a rigorous and systematic approach, there are important limitations to this study. Firstly, the data are based on a non-random sample of mostly health visitors and midwives in Scotland and the findings may not be generalisable to similar groups of participants outside this health setting. This has implications for our quantitative findings in particular. However, the collective maternity care experience of the sample strengthens the potential relevance of the identified strategies.

We used what we consider to be an under-utilised research method: concept maps. However, we used a hybrid version and we veered from the original methodology to meet the specific requirements of the study. Usually, statements within concept mapping are generated by participants themselves. In our study, the statements were generated from women’s experiences, facilitating a collaborative approach to identifying priority areas and strategies. This allowed participants to listen to and respond directly to women. We view this as a pragmatic approach that ensured congruence between methods and research questions and importantly, allowed the women’s perspectives to be acted upon.

This study was concerned with the experiences of disabled women with domestic abuse experiences. Although the focus groups were specifically about disabled women, participants spent surprisingly little time acknowledging disability related barriers. It might appear therefore that the issue of disability has become ‘lost’ within the dominant discourse of the paper regarding domestic abuse. However, we cannot account for the barriers, strategies and priorities that were identified – they originated from the women who took part in an earlier phase of the study and were built upon by the participants in the current phase. Our place as researchers is a conduit for the information and to report the results as honestly as possible. We do however recognise the paucity of direct, disability-related findings and suggest this as an area for further study.

Finally, while presenting the findings at conferences and during one of the focus groups, we have been challenged to consider the specificity of our findings. We have been asked whether many of the strategies are applicable to all women – not just those with disability and domestic abuse related issues. Our response is that we do not see this as a limitation. Providing accessible information about domestic abuse, adopting a non-judgemental attitude, creating safe spaces for disclosure and so on *are* important for all women who access maternity care. So getting these aspects of care right has implications beyond one specific group, to impact positively on the care of all women.

### Implications for maternity care practice

There are a number of implications for practice and research arising from this study. A significant strength is that we did not stop at the identification of barriers and identification of strategies. We continued on to investigate the feasibility of the identified strategies (as presented in Table 7). We saw this as an important step in beginning the implementation process and these findings provide a basis for further improvement work within the maternity care context. There is limited value in identifying theoretically interesting strategies if their feasibility is weak. In this study, the two priority areas relating to information and non-judgemental staff were also deemed to be among the most feasible. This is important if they are to become embedded in practice.

In our study, like many others, some health professionals were confident about asking about domestic abuse, but others need more support [57]. Training is an important factor in promoting health professionals’ confidence in addressing and responding to domestic abuse but our study highlights that, even despite training in domestic abuse, health and care professionals still find it difficult to ask and respond to domestic abuse. Moreover, we identified that professionals continue to hold misconceptions and stereotypical views around both disability and domestic abuse. We propose therefore that there is a need to review existing training given to staff – both in relation to disability and domestic abuse – in order to understand how training and education can be optimised as a means of changing culture and breaking down stigmatising views.

## Conclusions

Achievement of better health outcomes for women and new-born infants needs improvements in the quality of reproductive, maternal, and new-born care [68]. Equal access to good maternity care is essential to the health and wellbeing of all mothers and their babies. Timely, effective interventions during pregnancy can reduce the risk of preterm birth and perinatal loss, leading the World Health Organization (WHO) [69] to call for research that addresses the barriers to equitable maternity care. It is imperative that particular groups in society are not excluded from healthcare provision on the basis of biological, socio-economic factors or discrimination. But we know that often the inability of health and social care providers to recognise the heterogeneity of disabled people such as those also living with domestic abuse has a profound effect on their ability to access good healthcare. Within this context, our study has highlighted some important issues for disabled women affected by domestic abuse when accessing and using maternity services.

We began this paper with reference to equality of participation. Our enquiry has been about the ability of disabled women with domestic abuse experiences to access and utilise the maternity services that they need. Through our theoretical lens [41] we have identified the barriers that women face and importantly, the strategies that can be employed in order to overcome these. In many respects the study supports findings of previous studies regarding the barriers that women face. But we have provided new evidence on the perceived importance and feasibility of strategies to address these barriers. This is an important step in ensuring the practice-based acceptability and ease with which the improvement strategies might be implemented within the context of maternity care.

## Abbreviations

CIT: critical incident technique
NICE: National Institute for Health and Care Excellence
WHO: World Health Organization
